# Exploring Mitochondrial Localization of SARS-CoV-2 RNA by Padlock Assay: A Pilot Study in Human Placenta

**DOI:** 10.3390/ijms23042100

**Published:** 2022-02-14

**Authors:** Francesca Gabanella, Christian Barbato, Nicoletta Corbi, Marco Fiore, Carla Petrella, Marco de Vincentiis, Antonio Greco, Giampiero Ferraguti, Alessandro Corsi, Massimo Ralli, Irene Pecorella, Cira Di Gioia, Francesco Pecorini, Roberto Brunelli, Claudio Passananti, Antonio Minni, Maria Grazia Di Certo

**Affiliations:** 1 CNR-Institute of Biochemistry and Cell Biology, Department of Sense Organs, Sapienza University of Rome, Viale del Policlinico 155, 00161 Rome, Italy; francesca.gabanella@gmail.com (F.G.); christian.barbato@cnr.it (C.B.); marco.fiore@cnr.it (M.F.); carla.petrella@cnr.it (C.P.); 2 CNR-Institute of Molecular Biology and Pathology, Department of Molecular Medicine, Sapienza University of Rome, Viale Regina Elena 291, 00161 Rome, Italy; nicoletta.corbi@cnr.it; 3 Department of Sense Organs, Sapienza University of Rome, Viale del Policlinico 155, 00161 Rome, Italy; marco.devincentiis@uniroma1.it (M.d.V.); antonio.greco@uniroma1.it (A.G.); massimo.ralli@uniroma1.it (M.R.); 4 Department of Experimental Medicine, Sapienza University of Rome, Viale del Policlinico 155, 00161 Rome, Italy; giampiero.ferraguti@uniroma1.it; 5 Department of Molecular Medicine, Sapienza University of Rome, Viale Regina Elena 324, 00161 Rome, Italy; alessandro.corsi@uniroma1.it; 6 Department of Radiological, Oncological, and Pathological Sciences, Sapienza University of Rome, Viale del Policlinico 155, 00161 Rome, Italy; irene.pecorella@uniroma1.it (I.P.); cira.digioia@uniroma1.it (C.D.G.); 7 Department of Maternal and Child Health and Urological Sciences, Sapienza University of Rome, Viale del Policlinico 155, 00161 Rome, Italy; francesco.pecorini@uniroma1.it (F.P.); roberto.brunelli@uniroma1.it (R.B.)

**Keywords:** COVID-19, SARS-CoV-2 RNA, mitochondria, placenta, padlock

## Abstract

The ongoing COVID-19 pandemic dictated new priorities in biomedicine research. Severe acute respiratory syndrome coronavirus 2 (SARS-CoV-2), the causative agent of COVID-19, is a single-stranded positive-sense RNA virus. In this pilot study, we optimized our padlock assay to visualize genomic and subgenomic regions using formalin-fixed paraffin-embedded placental samples obtained from a confirmed case of COVID-19. SARS-CoV-2 RNA was localized in trophoblastic cells. We also checked the presence of the virion by immunolocalization of its glycoprotein spike. In addition, we imaged mitochondria of placental villi keeping in mind that the mitochondrion has been suggested as a potential residence of the SARS-CoV-2 genome. We observed a substantial overlapping of SARS-CoV-2 RNA and mitochondria in trophoblastic cells. This intriguing linkage correlated with an aberrant mitochondrial network. Overall, to the best of our knowledge, this is the first study that provides evidence of colocalization of the SARS-CoV-2 genome and mitochondria in SARS-CoV-2 infected tissue. These findings also support the notion that SARS-CoV-2 infection can reprogram mitochondrial activity in the highly specialized maternal–fetal interface.

## 1. Introduction

Severe acute respiratory syndrome coronavirus 2 (SARS-CoV-2), the infectious agent that causes COVID-19, is a type of single-stranded RNA virus that belongs to the beta coronavirus family [1,2]. Two-thirds of the viral genome include genes encoding for large polyproteins, which are then processed into 16 non-structural proteins (NSPs) by proteolytic cleavages. The remaining one-third of the genome consists of open reading frames (ORFs) for structural proteins such as spike, envelope, membrane, and nucleocapsid [2].

We aimed to evaluate the efficiency of our published padlock assay in visualizing SARS-CoV-2 RNA in formalin-fixed paraffin-embedded (FFPE) tissues. In fixed cells, padlock probes can target a selected RNA with near-single-molecule resolution [3,4,5,6]. This method provides intracellular localization maps of transcripts with high spatial resolution and sequence specificity. For our study, we took advantage of an archival FFPE tissue of the placenta from a pregnant woman with COVID-19. Contextually, we focused on placental mitochondria keeping in mind that increasing evidence points to the mitochondrion as an attractive target for both maintaining pregnancy and the SARS-CoV-2 life cycle [7,8,9]. Overall, to propagate itself and evade immune response, positive-sense RNA virus is prone to target mitochondrial dynamics [10,11]. Recently, an innovative biochemical approach showed a physical and functional interaction between SARS-CoV-2 and host mitochondria [12]. These findings were also supported by imaging approaches using SARS-CoV-2-infected cells [13]. However, the absence of images visualizing the interaction between SARS-CoV-2 and mitochondria in tissues from patients with COVID-19 remains a critical gap.

In this study, we showed that the padlock assay is a robust method to map the distribution of the SARS-CoV-2 genome in infected tissues. For the first time, we provided images visualizing the in situ colocalization of SARS-CoV-2 RNA and mitochondria in a patient. Furthermore, in the context of pregnancy complications, mitochondrial dysfunction appears to be a critical feature in the pathogenesis of preeclampsia [14]. Notably, pregnant women with COVID-19 are more likely to develop preeclampsia [15]. Collectively, this study opens a new scenario for SARS-CoV-2 investigations.

## 2. Results and Discussion

Exploring SARS-CoV-2 RNA in host cells not only assumes diagnostic significance but also allows us to critically contribute to advance the knowledge of the interaction between SARS-CoV-2 and human cells and, generally, of COVID-19. Viral RNA can be visualized on FFPE tissues using in situ hybridization (ISH) methods [16,17]. We recently optimized a padlock assay to visualize the RNA of interest in fixed cells. This method uses padlock probes combined with the rolling circle amplification (RCA) strategy [3,4,5]. The padlock assay is highly selective and efficient in identifying transcripts in fixed specimens [6]. In this study, we aimed to test and propose our padlock assay as an alternative approach to map SARS-CoV-2 localizations in infected tissues. SARS-CoV-2 genome consists of a positive-sense RNA with a size of about 30 kb [2]. We chose to target the viral RNA sequence encoding NSP7, a viral protein that we successfully tested in our experimental systems (data not shown). Next, we took advantage of an archival FFPE sample of a placenta at term from a woman in whom SARS-CoV-2 infection was diagnosed two months before labor. For comparison, we also evaluated additional FFPE samples of a placenta at term from a woman who gave birth before the COVID-19 pandemic. Although transient, the placenta is a fascinating organ that allows mother–fetus communication during pregnancy [18].

**Figure 1:**
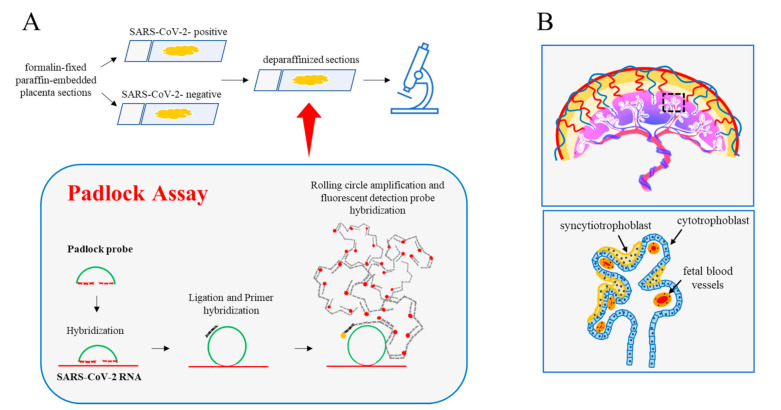
Padlock assay. (**A**) Diagram illustrating the main experimental steps of the padlock assay carried out in deparaffinized sections from the human placenta. Formalin-fixed paraffin-embedded sections (4 µm thickness) from the SARS-CoV-2 positive and SARS-CoV 2-negative placentas were first deparaffinized then processed for the padlock assay. A padlock probe was designed with the 5′- and 3′-terminal bases complementary to a selected sequence of SARS-CoV-2 RNA. The padlock probe was ligated and circularized with its complementary RNA template. A short DNA primer was used for initiating rolling circle amplification (RCA). In this way, the target sequence was converted in a long DNA amplicon with several copies of the padlock probe. Finally, amplicons became detectable by hybridization with the fluorophore-labeled probe. Sections were then analyzed by a fluorescence microscope. (**B**) (**top**) A schematic model of the anatomy of human placental villi. (**bottom**) Enlarged detail illustrating the major cellular components of the villus.

**Figure 2:**
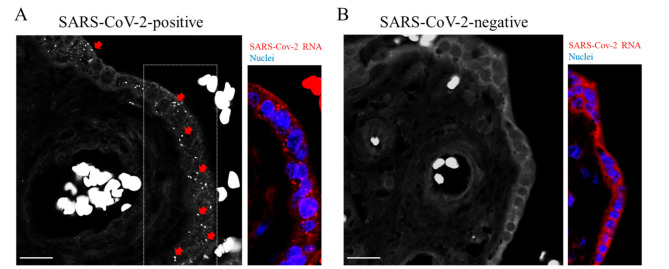
Detection of SARS-CoV-2 RNA. (**A**,**B**) Representative images of padlock assay targeting the SARS-CoV-2 RNA in sections from the placenta. (**A**) Genomic and subgenomic regions of SARS-CoV-2 were detectable following fluorescence microscopy in the SARS-CoV-2 positive placenta (red arrows and dots in a selected area of the corresponding color panel). Nuclei were labelled with DAPI (blue). (**B**) No specific signals were detectable in sections from the SARS-CoV-2 negative placenta. Scale bar, 20 µm.

Studies focusing on COVID-19 in pregnancy have shown the presence of the SARS-CoV-2 genome or proteins within the placental compartment [19,20]. A placental tropism seems justified by the expression of SARS-CoV-2 entry factors [21]. As schematically illustrated in Figure 1A, deparaffinized sections of both placentas were subjected to padlock assay, then imaged by a high-resolution fluorescence microscope. Figure 1B illustrates a simplified view of placental villi showing the trophoblast consisting of an outer layer of multinucleated syncytiotrophoblasts and an inner layer of mononucleated cytotrophoblasts that line the connective tissue core in which fetal blood vessels are placed [18]. By fluorescence microscope, placental villi were easily identified by the fluorescent background signal originating from tissue and by nuclei staining. Blood erythrocytes were detectable due to their peculiar and strong autofluorescence. The presence of SARS-CoV-2 RNA was revealed by RCA fluorescent dots (Figure 2A, white and red in black-and-white and colored images, respectively; Figure S1). Several dots were detectable in trophoblastic cells. This observation is in line with those in previous studies [19]. Padlock probe specificity was verified by monitoring a SARS-CoV-2 negative placenta (Figure 2B and Figure S1). In parallel, we checked the distribution of the spike protein, which is required for the binding of coronavirus to target cells (Figure S2). These findings not only validate the feasibility and efficiency of the padlock assay in detecting RNA of interest in FFPE tissues, but also provide an alternative and robust method to map SARS-CoV-2 RNA in infected tissues.

**Figure 3:**
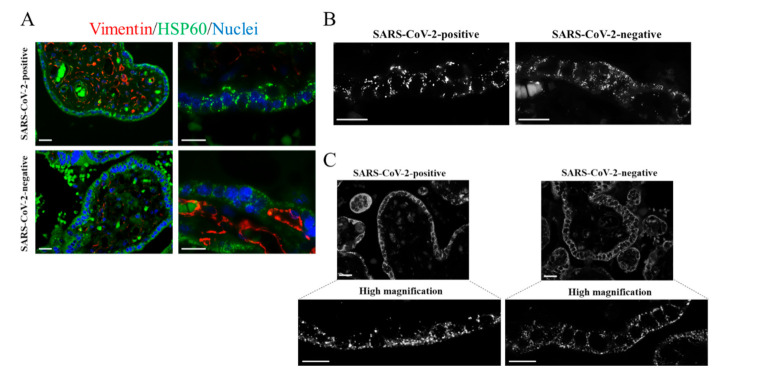
Immunostaining of placental mitochondria. (**A**–**C**) Representative images of the immunofluorescence analysis showing mitochondria in placental villi of the SARS-CoV-2 positive and SARS-CoV-2 negative placentas. The images were acquired with 40× or 100× oil objectives as indicated. (**A**) Placental sections were immunolabelled with the mitochondrial marker HSP60 (green). Vimentin immunostaining identified endothelial cells of blood vessels (red). Nuclei were labelled with DAPI (blue). (**B**) Representative images visualizing the mitochondrial marker HSP60, acquired with 100× oil objective, in villous trophoblastic cells. (**C**) Representative images, acquired with 100× oil objective, showing placental mitochondria immunolabelled with anti-COX IV. Scale bar, 20 µm.

In pregnant women, the specific clinical manifestation of SARS-CoV-2 infection remains unclear [19]. Only sporadic cases of vertical transmission were previously described [22]. Identifying molecular changes occurring in the maternal–fetal interface in pregnant women with COVID-19 represents a major challenge. Host cell mitochondria play a critical role in the SARS-CoV-2 life cycle. It was suggested that mitochondrial “hijacking” by SARS-CoV-2 might be a key component in the pathogenesis of COVID-19 [23]. On the other hand, placental mitochondria play a crucial role in maintaining pregnancy. Proper dynamics regulating the mitochondrial fission–fusion cycle prevent cell injury. Cytotrophoblast and syncytiotrophoblast cells exhibit distinct mitochondrial populations with different susceptibility to damage [24]. Cytotrophoblast mitochondria are relatively larger with lamellar cristae, whereas syncytiotrophoblast mitochondria are small and spherical with tubular cristae [24]. This may imply different mitochondrial susceptibility to damage between the different villous trophoblastic cell populations. Given the critical role of placental mitochondria, we were interested in imaging the mitochondria reticulum in a placenta with COVID-19. Mitochondria were labelled using two specific markers: anti-HSP60 and anti-COX IV, which are monoclonal and polyclonal antibodies, respectively. In combination with HSP60 antibody, we used anti-vimentin polyclonal antibody that marks stromal and endothelial cells. We found an aberrant mitochondrial reticulum in the SARS-CoV-2 positive placenta. As shown in Figure 3 (SARS-CoV-2 positive), mitochondria exhibited a polarized distribution and appeared strongly fused. In this one case of SARS-CoV-2 positive placenta, mitochondria appeared fused in both cytotrophoblast and syncytiotrophoblast. Moreover, several fragmented mitochondria were present. Conversely, SARS-CoV-2 negative placental villi exhibited a more regular mitochondrial reticulum. In this case, mitochondrial dynamic switched to a fission rather than a fusion event, coherent with the morphological changes occurring in the ageing placenta [25]. We obtained similar results with both mitochondrial markers (HSP60 and COX IV). In this context, most positive-strand RNA viruses replicate in association with cellular membranes derived from organelles including mitochondria [10]. Notably, SARS-CoV-2 was found to induce mitochondrial fusion, and this event was linked to the ability of the virus to prevent apoptosis [8,26].

**Figure 4:**
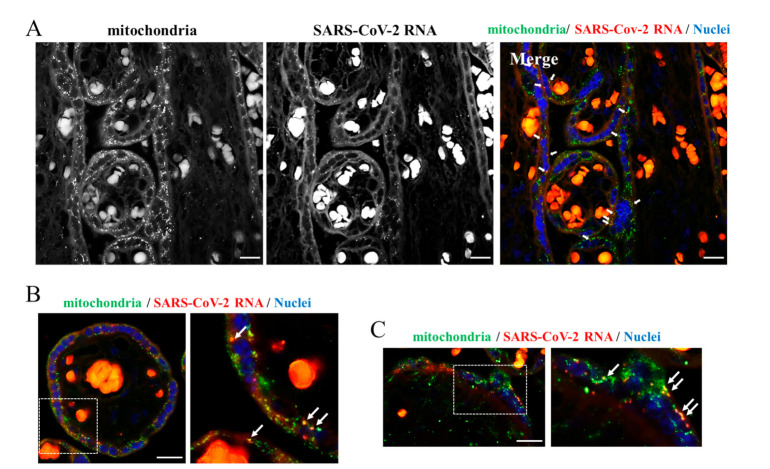
Dual staining of placenta sections. (**A**–**C**) Sections from the SARS-CoV-2 positive placenta were subjected to a combination of the padlock and immunofluorescence assays to visualize the SARS-CoV-2 RNA (red in the color images) and mitochondria (green in the color images). In panel (**A**,**B**), HSP60 was used as mitochondrial marker. In panel (**C**), COX IV was used as mitochondrial marker (green in the color panels). SARS-CoV-2 RNA (red in the color panels) results partially overlapped with both mitochondrial markers in the Merge panel (yellow dots). In panel (**B**,**C**), a higher magnification of a selected region is shown. Scale bar, 20 µm.

Similarly, the genomic and subgenomic regions of SARS-CoV-2 were predicted to localize in mitochondria of the host cell [23]. This notion remains a prediction since experimental images of this event in tissues with COVID-19 are so far non-existent. The availability of a placenta from a woman with COVID-19 prompted us to explore this intriguing aspect. We carried out an immunofluorescence analysis combined with the padlock assay. In our experience, this combined approach provides high-quality colocalization images even though it reduces the efficiency of both the immunofluorescence and padlock assay. As shown in Figure 4, several dots corresponding to SARS-CoV-2 RNA clearly overlapped with placental mitochondria. We are the first to provide images highlighting physical contact between the SARS-CoV-2 RNA and host cell mitochondria. It is fascinating that this first evidence was produced in a unique organ such as the placenta. In this regard, images on the mitochondrial engagement by SARS-CoV-2 RNA can provide a mechanistic and clinical interpretation of preeclampsia in women with COVID-19. This pilot study prompts us to further explore the relationship between SARS-CoV-2 RNA and mitochondria and further upgrade our imaging approach based on padlock probes, to contribute to the advance in the knowledge of the pathogenesis of COVID-19.

## 3. Materials and Methods

### 3.1. Placental Tissue Samples

Archival FFPE tissue samples from two placentas at term were included in this pilot study. One of the placentas was from a 30-year-old woman who gave birth in 2021 and in whom SARS-CoV-2 infection was diagnosed two months before labor. The other placenta was from a 36-year-old woman who gave birth in 2018, i.e., before the COVID-19 pandemic (https://www.who.int/emergencies/disease-outbreak-news/item/2020-DON229, accessed on 3 January 2022), which was not tested for SARS-CoV-2 infection. No other clinical data were available. We used 4 µm thick sections obtained from the paraffin blocks loaded onto positively charged glass slides for the immunofluorescence studies. The study protocol conformed to the Declaration of Helsinki and its later amendments and was approved by the internal Institutional Review Board (Ethical Committee of Sapienza University of Rome, Rome, Italy, Policlinico Umberto I, approval number: 6536).

### 3.2. Antibodies and Reagents

We used anti-HSP60 mouse monoclonal antibody (Santa Cruz Biotechnology, Dallas, TX, USA; work dilution for immunofluorescence, 1:100); anti-COX IV rabbit polyclonal antibody (Millipore, Burlington, MA, USA; 1:200); anti-spike mouse monoclonal antibody (Santa Cruz Biotechnology; 1:100); anti-Vimentin rabbit monoclonal antibody (Abcam, Cambridge, UK; 1:150); and Alexa-Fluor-488- or Alexa-Fluor-594-conjugated secondary antibodies (Life Technologies, Carlsbad, CA, USA; 1:200).

### 3.3. Immunofluorescence Analysis

Paraffin-embedded sections were dewaxed by 2 changes of xylene, 30 min each. After hydration in graded ethanol solutions (100%, 95%, 90%, 80%, 70%, 50%, and 30% ethanol, for 2 min each), sections were incubated for 30 min at 95 °C with antigen retrieval solution (10 mM sodium citrate, 0.05% Tween 20, pH 6.0). Slides were rinsed in PBS/0.1% Tween 20; blocked with 1% BSA in PBS for 1 h at room temperature; incubated at 4 °C overnight using the appropriate primary antibodies; washed three times in PBS/0.1% Tween 20; and incubated with the appropriate secondary antibodies. Slides were mounted with ProLong with DAPI (Thermo Fisher Scientific, Waltham, MA, USA) and examined by an epifluorescence microscope (Olympus BX53; Milan, Italy) equipped with a SPOT RT3 camera. Images were merged using the image analysis software IAS 2000 (Delta Sistemi, Alessandria, Italy).

### 3.4. Padlock Assay

Paraffin-embedded sections were dewaxed by treating with xylene overnight. Sections were then hydrated in 100%, 95%, 90%, 80%, 70%, 50%, and 30% ethanol for 2 min each and rinsed in distilled water. To preserve RNA during the following procedure, sections were fixed again in 4% formaldehyde for 20 min. Samples were incubated for 30 min at 80 °C in the unmasking solution (10 mM sodium citrate, 0.05% Tween 20, pH 6.0), then allowed to cool to room temperature for 10 min. After rinsing with PBS, the slides were processed for the padlock assay. Sections were incubated overnight at 37 °C with the specific padlock probe to the target mRNA. The reaction was carried out in 50 µL of a solution containing 7.5 µL of phosphorylated padlock probe (10 µM), 2.5 µL of DTT (100 mM), 1.25 µL of RiboLock RNase inhibitor (40 U µL^−1^), and 38.75 µL of DEPC-treated H_2_O. After a wash in PBS/0.01% Tween 20, the samples were incubated for 2 h at 37 °C in 50 µL with the ligation reaction mixture (1× SplintR ligase buffer, 2.5 U µL^−1^ SplintR ligase, and 1 U µL^−1^ RiboLock RNase inhibitor). Subsequently, the sections were washed in PBS/0.01% Tween 20 and incubated for 1 h at 37 °C with the RCA primer mixture (0.2 µM RCA primer, 1× SSC, 10% formamide, 5 mM DTT, and 0.5 U µL^−1^ RiboLock RNase inhibitor). The RCA reaction was then carried out for 2 h at 37 °C. The sample was incubated with 50 µL of a mixture containing 5 µL of 10× phi29 DNA polymerase reaction buffer, 2.5 µL of phi29 DNA polymerase (10 U µL^−1^), 15 µL of dNTPs (10 mM of each dATP, dCTP, dGTP, and dTTP), 1.25 µL of RiboLock RNase inhibitor (40 U µL^−1^), and 26.25 µL of DEPC-treated H_2_O. The incubation was followed by a wash in PBS/0.01% Tween 20. Finally, slides were incubated with a mixture containing 100 nM AlexaFluor-595-labelled detection probe, 2× SSC, and 15% formamide for 30 min at 37 °C, then washed in PBS/0.01% Tween 20 three times for 5 min each. Slides were mounted with ProLong with DAPI (Thermo Fisher Scientific, Waltham, MA, USA) and examined by a conventional epifluorescence microscope (Olympus BX53; Milan, Italy) equipped with a SPOT RT3 camera. Images were merged using the image analysis software IAS 2000 (Delta Sistemi, Alessandria, Italy). When the padlock assay was combined with immunofluorescence analysis, slides were processed for the padlock assay, incubated with the appropriate primary antibodies, washed three times in PBS/0.1% Tween 20, then incubated with the appropriate secondary antibodies.

The oligos used in this study are indicated in the Supplementary Material.

## Data Availability

The authors confirm that the data supporting the findings of this study are available within the article.

## Supplementary Materials

The following supporting information can be downloaded at: https://www.mdpi.com/article/10.3390/ijms23042100/s1.
