# Supplementary material for: Exploring Mitochondrial Localization of SARS-CoV-2 RNA by Padlock Assay: A Pilot Study in Human Placenta
Source: Int J Mol Sci. 2022 Feb 14;23(4):2100. doi: 10.3390/ijms23042100 (PMC8875563; doi:10.3390/ijms23042100)
Supplement: Supplementary file 1 [file ijms-23-02100-s001.zip › ijms-1564601-supplementary.pdf]

Table S1. Oligos used in this study.

Padlock Probe NSP7	5’ACATTGAGCCCACATTTTTTTCTCAATTCTGCTACTTTACTACCTCAATTCTGCTACTG TACTACTTTTTTCATTGTGTAACTGGA 3’
RCA Primer	AGTACAGTAGCAGAATTGAG
AlexaFluor 595-labelled probe	CTCAATTCTGCTACTTTACTAC

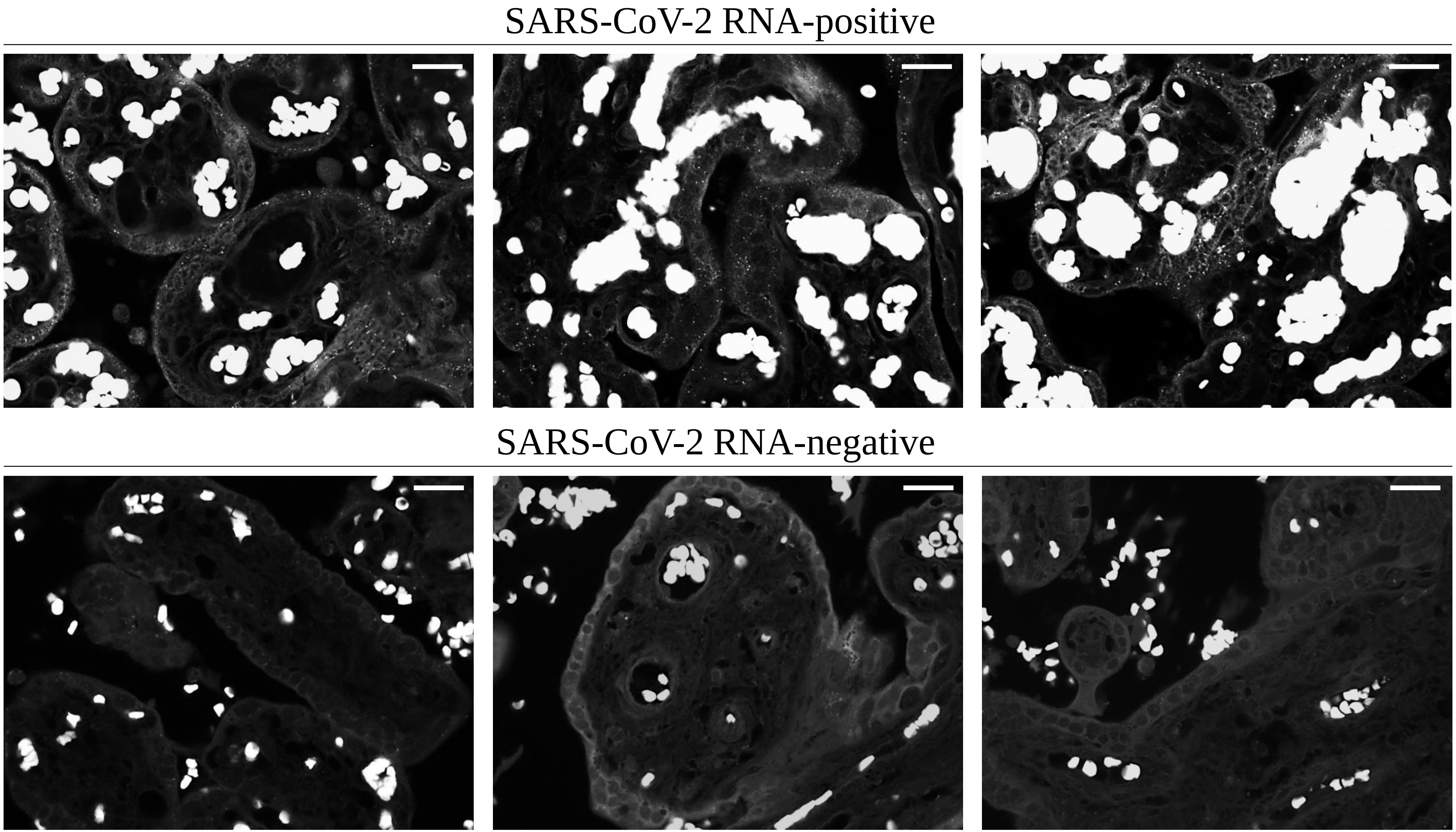

Figure S1. SARS-CoV-2 RNA detection by Padlock Assay. Microscope fields (*black and white*) of padlock assay targeting the SARS-CoV-2 RNA in SARS-CoV-2-positive placenta. No specific signals are detectable in sections from a SARS-CoV-2-negative placenta. Scale bar, 20 μm.

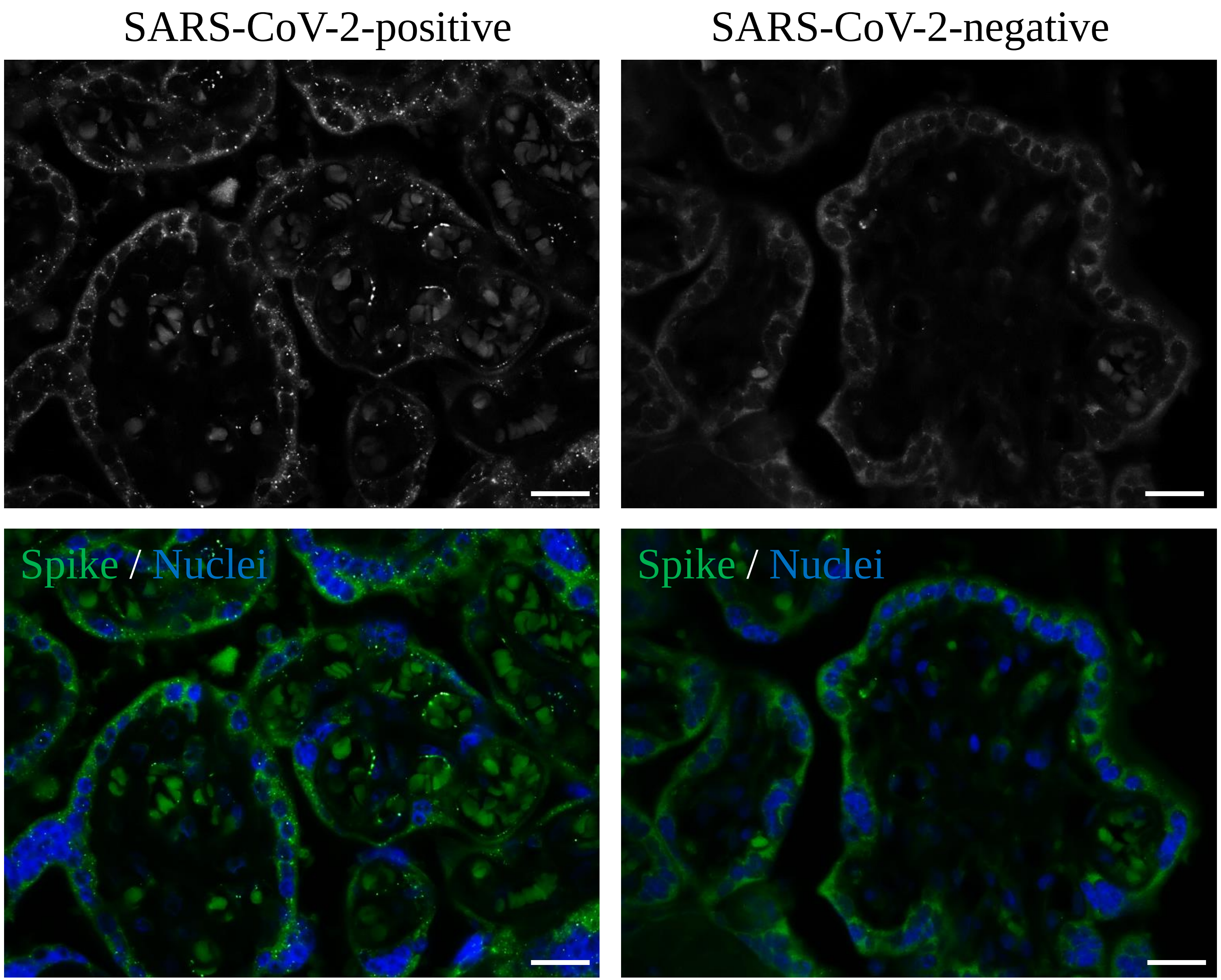

Figure S2. Immunostaining of spike protein. Representative images of the immunofluorescence analysis showing the distribution of spike protein in deparaffinized sections of SARS-CoV-2-positive placenta. No specific signals are detectable in sections from SARS-CoV-2-negative placenta. Nuclei were labelled with DAPI (blue). (both *black and white* and *coloured* microscope fields). Scale bar, 20 µm.
